# Model of the SARS-CoV-2 Virus for Development of a DNA-Modified, Surface-Enhanced Raman Spectroscopy Sensor with a Novel Hybrid Plasmonic Platform in Sandwich Mode

**DOI:** 10.3390/bios12090768

**Published:** 2022-09-19

**Authors:** Mariia V. Samodelova, Olesya O. Kapitanova, Nadezda F. Meshcheryakova, Sergey. M. Novikov, Nikita R. Yarenkov, Oleg A. Streletskii, Dmitry I. Yakubovsky, Fedor I. Grabovenko, Gleb A. Zhdanov, Aleksey V. Arsenin, Valentyn S. Volkov, Elena G. Zavyalova, Irina A. Veselova, Maria I. Zvereva

**Affiliations:** 1 Faculty of Chemistry, Lomonosov Moscow State University, Leninskie Gory 1, 119991 Moscow, Russia; 2 Center for Photonics and 2D Materials, Moscow Institute of Physics and Technology, 141700 Dolgoprudny, Russia; 3 Faculty of Physics, Lomonosov Moscow State University, Leninskie Gory 1, 119991 Moscow, Russia

**Keywords:** surface-enhanced Raman scattering (SERS), aptasensor, SARS-CoV-2 virus

## Abstract

The recent severe acute respiratory syndrome coronavirus 2 (SARS-CoV-2) infection has posed a great challenge for the development of ultra-fast methods for virus identification based on sensor principles. We created a structure modeling surface and size of the SARS-CoV-2 virus and used it in comparison with the standard antigen SARS-CoV-2—the receptor-binding domain (RBD) of the S-protein of the envelope of the SARS-CoV-2 virus from the Wuhan strain—for the development of detection of coronaviruses using a DNA-modified, surface-enhanced Raman scattering (SERS)-based aptasensor in sandwich mode: a primary aptamer attached to the plasmonic surface—RBD-covered Ag nanoparticle—the Cy3-labeled secondary aptamer. Fabricated novel hybrid plasmonic structures based on “Ag mirror-SiO_2_-nanostructured Ag” demonstrate sensitivity for the detection of investigated analytes due to the combination of localized surface plasmons in nanostructured silver surface and the gap surface plasmons in a thin dielectric layer of SiO_2_ between silver layers. A specific SERS signal has been obtained from SERS-active compounds with RBD-specific DNA aptamers that selectively bind to the S protein of synthetic virion (dissociation constants of DNA-aptamer complexes with protein in the range of 10 nM). The purpose of the study is to systematically analyze the combination of components in an aptamer-based sandwich system. A developed virus size simulating silver particles adsorbed on an aptamer-coated sensor provided a signal different from free RBD. The data obtained are consistent with the theory of signal amplification depending on the distance of the active compound from the amplifying surface and the nature of such a compound. The ability to detect the target virus due to specific interaction with such DNA is quantitatively controlled by the degree of the quenching SERS signal from the labeled compound. Developed indicator sandwich-type systems demonstrate high stability. Such a platform does not require special permissions to work with viruses. Therefore, our approach creates the promising basis for fostering the practical application of ultra-fast, amplification-free methods for detecting coronaviruses based on SARS-CoV-2.

## 1. Introduction

Surface-enhanced Raman scattering (SERS)-based sensors for viral detection are very attractive, combining high recognition ability and sensitivity with a rapidness of the analysis. The ultimate specificity can be achieved for low-molecular compounds, providing identification via the unique Raman spectrum of the compound. In this regard, the development of novel improved SERS-active substrates is of high interest [1]. The development and implementation of reliable methods for the synthesis/fabrication of uniform, reproducible, low-cost SERS substrates with a high enhanced factor and quantitative response within specification limits is being conducted. Thus, the natural reed leaves, without any special pretreatment, coated by silver, have been utilized as a self-assembled plasmonic structure for ultra-sensitive crystal violet trace detection with sensitivity as low as 10^−13^ M using a novel natural surface-enhanced fluorescence [2]. Recently, a scalable design of SERS substrate based on a biopolymer of free-standing chitosan film, silver nanoparticles, and graphene oxide has been demonstrated [3]. The developed plasmon hybrid structures were assessed by their SERS-sensitivity, reproducibility, stability, and quenching capability. The fabricated plasmonic polymer nanocomposite offered a limit of detection for R6G down to 100 pM. Using the 3D jet writing technique, biologically inert poly(lactic-co-glycolic acid) tessellated scaffolds containing fluorescent polymers and/or SERS-encoded gold nanostars have been fabricated [4]. Such a hybrid scaffold provides efficient work with high-molecule volumetric objects such as live cells and offers sufficient optical transparency for both fluorescence and SERS imaging. The novel hybrid metal-dielectric-metal sandwiched structures [5,6] demonstrate high sensitivity and reproducibility of the SERS signal [7,8]. In this geometry, the overall enhancement factor principally benefits from the combination of two main mechanisms. The first one is associated with the excitation of localized surface plasmon resonance (SPR) in gold/silver clusters. The second one is due to the gap surface plasmons’ excitation in a thin dielectric layer between the metal mirror and corrugated gold/silver layers. SERS has attracted much attention for its potential in multiplexed sensing. SERS is an optical molecular finger-printing technique that has the ability to resolve analytes from within mixtures [9]. However, the biological macromolecules are composed of the same set of building blocks—amino acids, nucleotides, and sugars, that creates difficulties for SERS detection based on biopolymer nature [10]. The specific determination of one protein in a mixture of dozens of other macromolecules is challenging [11,12,13]. Speaking of viruses, reported Raman spectra of the S protein of SARS-CoV-2 are not the same [14,15,16,17], and Raman spectra of influenza virions are rather different [14,15,18,19]. The differences in spectra could be due to variations of impurities in biological samples or different SERS-active substrates that enhanced Raman spectra of different groups in macromolecules. In both cases, their application for real clinical samples is questionable.

Recognizing molecules, like antibodies or aptamers, can be used to provide the specificity of virus detection along with an increase in the limit of detection [11,12,13]. The most common approach is to decorate a SERS-active surface with recognizing molecules enhancing the local concentration of the target of interest near the surface [15,20,21,22,23]. A complementary approach uses labeled recognizing molecules to trace the spectrum of the artificial molecule that is detectable in the nanomolar range of concentrations [22,23,24,25]. The typical Raman active labels used for virus detection are fluorescent dyes like Cyanine 3 (Cy3), Cyanine 5.5 (Cy5.5), Bodipy FL (BDP FL), and Rhodamine Red-X (RRX) [22,23,24,25]. Labeled recognizing molecules are either approaching the surface due to sandwich-like ternary complexes [22,23] or retiring from the surface due to binding with viral particles [24,25]. In both cases, the Q-factor and stability in biological fluids are the key parameters that are to be considered. Silver nanoislands and lithography-based substrates were used for the sandwich-like complex aptamer1-virus-aptamer2 [22,23]. The first one provided nice SERS spectra for ternary complexes with Cy3 and BDP FL-labeled aptamers [22]; however, the stability of the nanostructured surface in biological fluids was revealed to be suboptimal (authors’ unpublished data). Lithography-based substrates are usually stable in various media; however, the SERS spectra of ternary complexes with Cy5.5-labeled aptamers were not acquired [23]. The detected differences in spectra could be attributed to surface-enhanced fluorescence of utilized model dye.

There is a growing interest in aptamer-based assay methods, without signal amplification, due to their low cost, having high stability, being easy to modify, and, in some cases, they have better specificity and affinity [26]. Among them, an electrochemical aptasensor for the determination of the SARS-CoV-2 receptor-binding domain in human saliva samples demonstrated high sensitivity (down to 7.0 pM) and accuracy [27]. Photonic biosensing technologies that have been explored for possible use in SARS-CoV-2 serology include SPR [28,29] for not only the detection of antibodies but also the profiling of binding kinetics of the complete polyclonal antibody response against the receptor-binding domain. Moreover, the asymmetric Mach–Zehnder Interferometer enables multiplex and ultra-sensitive detection of antibodies that target the viral antigens:spike protein, the receptor-binding domain, and the nucleocapsid protein [30]. SERS-based aptasensors have been used to detect both SARS-CoV-2 spike protein [31] and the whole virus of SARS-CoV-2 [18,23,25,32], providing good accuracy and satisfactory performance for SARS-CoV-2 determination in good agreement with RT-qPCR results [33]. However, the limit of detection of the sensors is still higher than RT-qPCR. Increasing the sensitivity of the method will increase the reliability of the results of the virus detection. The research work requires a good model system for the sensor optimization. The RBD protein per se cannot be used as a model, as the viruses contain dozens or hundreds of surface proteins that bind the solid substrates in multiple points. Therefore, the exemplary limit of detection of the virus is typically lower compared to those for the recombinant protein [18,23,25,31,32]. Production of virus-like particles (VLP) requires genetic engineering, and the stability of these membrane structures is to be traced during storage. The production of VLP requires laboratory space organized according to special strict safety requirements. In our work, we proposed a simplified imitation of the virus surface produced by the adsorption of RBD protein onto silver nanoparticles. We optimized the conditions to make the nanoparticles recognizable by the aptamers to RBD and storable for several weeks. This model of synthetic virion was used to screen SERS-substrates, revealing the mirror silver-dielectric-nanostructured silver sandwiched plasmonic nanostructures as a promising tool for SARS-CoV-2 detection. A developed indicator system for SARS-CoV-2 virions demonstrates applicability of the SERS approach for the selective detection of coronavirus-2 infection.

## 2. Materials and Methods

### 2.1. Oligonucleotides and Some Materials

Inorganic salts and buffer solutions, as well as bovine serum albumin, were purchased from Sigma-Aldrich (New York, NY, USA).

The following oligonucleotides were studied. Selected in lab [34], Found-SH (SH-CACCGCTTTTGCCTTTTGGGGACGGATATAGGGAAACACGATA GAATCCGAACAGCACC), and complementary to Found—CompFound-SH (SH-CACCGCTTTTGCCTTTTGGGGACGGATAGGTGCTGTTCGGATTCTATCGTGTTTCCCTA)—were kindly synthesized by the group of Timofei Zatsepin (Moscow, Russia). RBD-1C selected in [35] in two forms: RBD-1C-Cy3(Cy3-5′-CAGCACCGACCTTGTGCTTTGGGAGTGCTGGTCCAAGGGCGTTAATGGACA-3′) and biotinylated aptamer RBD-1C (biotin-5′-CAGCACCGACCTTGTGCTTTGGGAGTGCTGGTCCAAGGGCGTTAATGGACA-3′) from Eurogene (Moscow, Russia). The receptor-binding domain of the S-protein of SARS-CoV-2 (further referred to as RBD) was from [36] (for details of aptamer preparation and binding experiments see in supplementary material citation, Figure S1).

### 2.2. Synthesis of the Model of SARS-CoV-2 Virions

Silver nanoparticles (NP) were obtained by mixing 18 mL of a solution of 3.3 mM NaOH and 2.6 mM NH_2_OH∙HCl with 2 mL of 10 mM AgNO_3_. The solution of AgNO_3_ was injected for 10 s with a subsequent stirring for 1 h at room temperature.

RBD, expressed in Chinese hamster ovary cell line [36], was obtained in a concentration of 4.7 mg/mL. An amount of 6.4 µL of RBD protein solution was added to 1 mL of silver nanoparticles, providing a final concentration of the protein of 1 µM. The mixture was incubated at room temperature for 30 min. Unreacted protein was removed by centrifugation for 10 min at 10 rpm. The RBD-coated silver nanoparticles (further referred to as RBD NP) were resuspended in 1 mL of buffer with 10 mM Tris-HCl pH 7.5, 140 mM NaNO_3_, and 10 mM KNO_3_. Bovine serum albumin (further referred to as BSA) coated silver nanoparticles (further referred to as BSA NP) were obtained following the same protocol using 10 µM solution of BSA instead of RBD protein.

### 2.3. Characterization of Nanoparticles Coated with RBD Protein

A dynamic light-scattering instrument ZetasizerNano ZS (Malvern, Worcestershire, UK) was used to estimate the size and ζ-potential of nanoparticles.

The ability to bind aptamers to RBD was estimated using biolayer interferometry (Blitz equipment from ForteBio, Dallas, TX, USA). The sensor was hydrated in water, and then it was incubated with 1 μM solution of biotinylated aptamer RBD-1C for 2 min. The baseline was acquired in the binding buffer (10 mM Tris-HCl pH 7.5, 140 mM NaNO_3_, 10 mM KNO_3_) for 30 s. Then, the sensor was placed into RBD NP or BSA NP solutions for 200 s. The 2×, 4×, and 8× dilutions of the nanoparticle samples were obtained in the binding buffer. The dissociation was performed in the binding buffer.

### 2.4. SERS-Active Aptasensor Platform

The idea of the aptasensor platform is a formation of sandwich-like complexes on the silver nanostructured surface. The primary aptamer was modified with a thiol-group providing stable linkage between the plasmonic nanostructured silver surface and the aptamer. Primary aptamers bound RBD NP. Secondary aptamers with a Raman-active label (Cy3) formed complexes with RBD NP immobilized on the surface. The detailed protocol was as following: (1) SERS substrate was incubated in 20 µL of a 20 nM solution of thiol-modified aptamer in PBS for 15 min; (2) the substrate was rinsed with PBS; (3) the substrate was incubated in 20 µL of RBD NP (or BSA NP in the control experiment) solution for 5 min; (4) the substrate was rinsed with PBS; (5) the substrate was incubated in 20 µL of a 200 nM solution of labeled aptamer in PBS for 5 min; (6) the substrate was rinsed with water; (7) the substrate was dried in air.

### 2.5. SERS-Based Substrates

SERS platforms were prepared by method as previously described [37]. The fabrication of plasmonic substrates was performed in a preliminary evacuated chamber (base pressure 10^−5^ Torr). Then, the chamber was filled with Ar gas. The total pressure in the preparation chamber was 5 × 10^−4^ Torr and the temperature ~300 K. The 100 nm layer of Ag (99.999%) was deposited on silicon substrate, a 3 nm Ti adhesion interlayer over Ag and 50 nm layer of SiO_2_. A thin titanium layer was introduced, as it promotes adhesion, followed by deposited SiO_2_ to the silver layer. Then, a layer of 20 nm silver (99.999%) was deposited on the top of SiO_2_. These layers were deposited in one regime by electron beam evaporation. Then, the substrates were treated by an Ar ion beam with an energy of 150 eV and dose of 4 × 10^16^ ion/cm^2^. The angle between the Ar beam and substrate was 45°. A Hall-effect ion source with a cold hollow cathode Klan 53-M (Platar Corp.) generated an Ar ion beam. An ion beam modification of silver film allows for the fabrication of plasmonic nanostructured surfaces.

### 2.6. Characterization of SERS-Based Substrates

The surface morphology of the deposited films was investigated using a scanning electron microscope (SEM) JEOL JSM-7001F (JEOL Ltd., Tokyo, Japan).

Raman spectroscopy and SERS studies were carried out using the Enspectr R532 analyzer (Enhanced Spectrometry, San Jose, CA, USA) with a solid-state laser (wavelength—532 nm, 50 MW). All spectra were collected with the working laser spot diameter of ca. 2 µm through a × 40 objective. The specific power of the laser was set at 0.3 mW, and the accumulation time was 5 s with 10 accumulations. The obtained Raman scattering spectra were fitted using the pseudo-Voigt functions. Measurements were carried out in the range of 200–2000 cm^–1^ using 3rd-order baseline subtraction.

## 3. Results and Discussion

**Figure 1:**
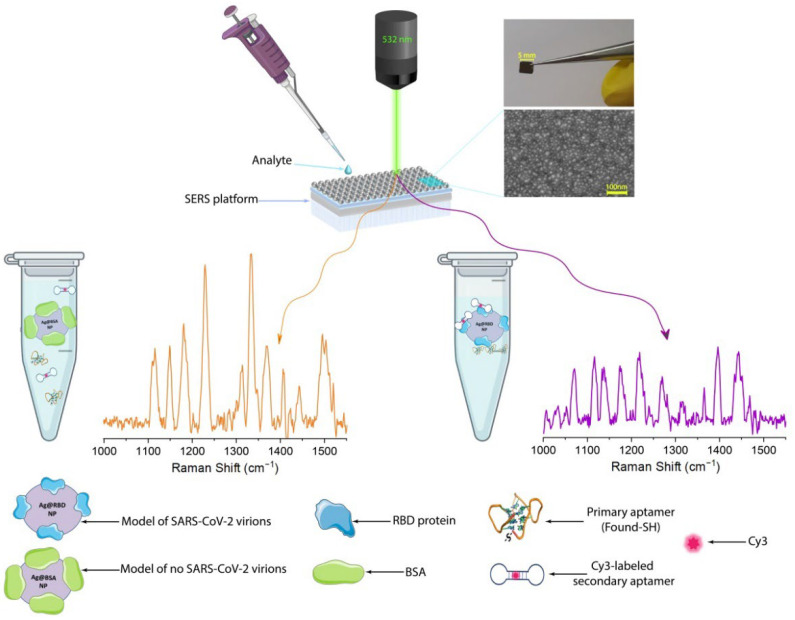
Scheme of the developed indicator system for SARS-CoV-2 virions’ detection using the SERS approach.

In this work, we have developed an indicator system for the recognition of SARS-CoV-2 virions in the future. We have determined optimal conditions for obtaining the artificial model of SARS-CoV-2 virions through a specific binding of a Cy3-labeled secondary aptamer and a primary aptamer with RBD-covered Ag nanoparticles (both the primary and secondary aptamers interact with the protein with approximately the same efficiency). In parallel, nonspecific interaction of both aptamers to BSA-covered Ag nanoparticles (Table 1), allows for the creation of a model without SARS-CoV-2 virions. A developed indicator system demonstrates applicability of the SERS approach for the selective detection of coronavirus-2 infection through the control of the distance between the SERS platform and the dye moiety. Figure 1 illustrates the platform of the SERS-active aptasensor using hybrid plasmonic substrates for the quantitative detection of coronaviruses. The self-assembled RBD nanoparticles blocked the direct adsorption of the Cy3-labeled secondary aptamer on a SERS-active nanostructured surface due to the specific interaction between all components in the assay leading to a strong quenching Raman signal of Cy3 (Figure 1).

### 3.1. Fabrication and Characterization of Model of SARS-CoV-2 Virions

**Figure 2:**
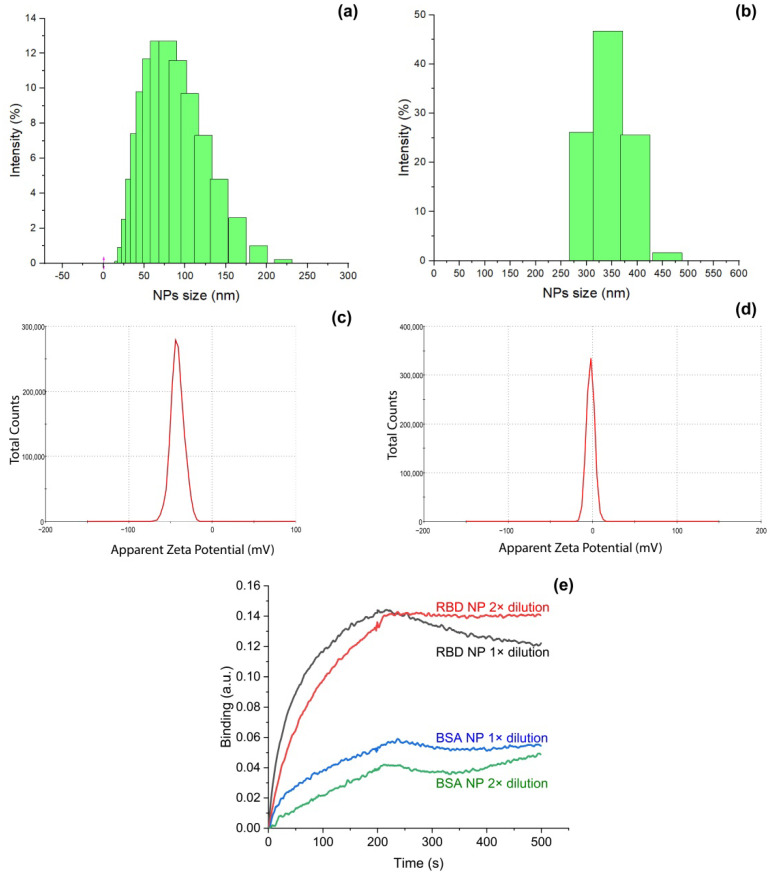
The size distribution for unmodified nanoparticles (**a**) and RBD-coated nanoparticles (**b**) estimated with dynamic light scattering. ζ-potential of unmodified nanoparticles (**c**) and RBD-coated nanoparticles (**d**) estimated with dynamic light scattering. (**e**) Interaction between aptamer RBD-1C and silver nanoparticles coated with RBD or BSA proteins.

Silver nanoparticles adsorb proteins, readily forming stable coatings [38]. We optimized the conditions for RBD and BSA proteins that were used as coating for silver nanoparticles with a diameter of 80 nm. Stable nanoparticles were obtained after incubation with 1 µM solution of RBD and 10 µM solution of BSA. An amount of 1 µM solution of BSA provided aggregates of nanoparticles during the storage, indicating that the protein coating was not completed. The excess of the proteins was removed by the centrifugation in both cases. The estimated concentration of the protein-coated nanoparticles was 10^10^ particles per mL. The optimized protocol provided nanoparticles that are stable for more than 6 weeks of storage at +4 °C. Unmodified NP and RBD NP were studied with dynamic light scattering. RBD NP was shown to have a significantly increased size compared to uncoated nanoparticles. The diameter of RBD NP was 320 ± 30 nm, whereas the diameter of NP was 80 ± 30 nm (Figure 2a,b). The size of RBD NP is overestimated, as protein coating decreases the mobility of silver nanoparticles. Similarly, ζ-potential was altered significantly from −42 ± 8 mV for NP to −2 ± 5 mV for RBD NP (Figure 2c,d), indicating significant changes of the surface of silver nanoparticles.

We used DNA aptamer RBD-1C selected by Song et al. [35] as a recognition molecule; this aptamer has dissociation constants in the range of 0.8–5.8 nM for the complexes with recombinant RBD protein [39]. Adsorbed RBD retained the ability of binding DNA aptamers as shown by biolayer interferometry (Figure 2e). The binding of BSA NP to DNA was 3–4 times lower, indicating a low level of nonspecific interaction. The overall dataset confirmed the formation of RBD-coated nanoparticles with a size close to the size of SARS-CoV-2 virions; RBD NPs are capable of binding DNA aptamers to RBD protein. This model system was used in further SERS experiments. Proposed particles are simple in preparation, stable during the storage, and composed of one type of protein, providing low non-specific binding.

### 3.2. Characterization of the Hybrid Plasmonic Sandwiched Structures

**Figure 3:**
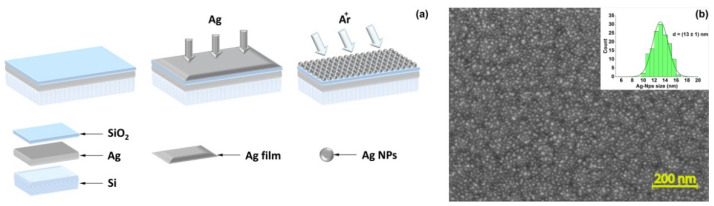
(**a**) Scheme of the fabrication of the hybrid plasmonic sandwiched structure: films of Ag, SiO_2_, Ag stepwise formed by electron beam sputtering with the further formation of a plasmonic silver-based structure through the Ar ion beam treatment of the top Ag film. (**b**) A typical SEM image of the sensor element decorated by Ag nanoparticles. The inset shows a size distribution of silver nanoparticles on the sensor element.

First, we obtained an efficient plasmonic sandwiched structure as the SERS-active platform. The surface of the sensor element is represented with a multilayered structure based on subsequently coated silver mirror film, dielectric film, and Ag nanoparticles, further referred to as film-Ag/SiO_2_/nano-Ag NPs (Figure 3a). As noted above, the amplification of the Raman signal for sandwiches in comparison with one-contained-layer nanostructured silver surface is associated with the excitation of both gap plasmons in the SiO_2_ layer and local surface plasmon resonances in nano-Ag NPs [5,6,7,8]. Moreover, the back reflection from the bottom Au layer gives rise to a stronger absorbance and, therefore, the enhancement factor (EF) since the excitation beam passes through the top film twice. The morphology of the obtained substrates was studied by scanning electron microscopy (SEM) (Figure 3b). As it is shown on an SEM image, silver nanoparticles are evenly distributed and the plasmonic surface has a reproducible structure. The average size of a particle was 13 ± 1 nm for 150 particles (Figure 3b, the inset).

The maximum of the plasmonic band of the SERS-active substrate appears at 425 nm (Figure S2). Moreover, a wide plasmonic peak covers the wavelength range from the blue to the IR regions of the spectrum. To evaluate the SERS activity of the designed hybrid substrate, first, we analyzed a model dye Cy3. The EF of the obtained hybrid surfaces was calculated according to the following equation [40]:

(1) $$\mathrm{EF}=\frac{I_{\mathrm{SERS}}\;{\;C}_{\mathrm{ref}}}{I_{\mathrm{ref}}{\;C}_{\mathrm{SERS}}}$$

where I_ref_ and I_SERS_ are the band’s intensities of the Raman scattering and SERS at 1397 cm^−1^ for Cy3; C_ref_ = 1 × 10^−2^ M and C_SERS_ = 1 × 10^−6^ M—concentrations of the analytes whose signals correspond to Raman scattering and SERS, respectively. Corresponding SERS and Raman spectra of Cy3 are in Figures S3 and S4 of the supplementary information. The EF of the resulting surface was 9.5 × 10^4^ for Cy3. Moreover, highly reproducible morphology of developed hybrid plasmonic substrates results in a good analytical signal reproducibility from point-to-point of the sample with the relative standard deviation (RSD) for the EF value as low as 7.37%.

### 3.3. SERS-Active Aptasensor Platform for the Detection of Model of SARS-CoV-2 Virions Using Hybrid Plasmonic Substrates

**Figure 4:**
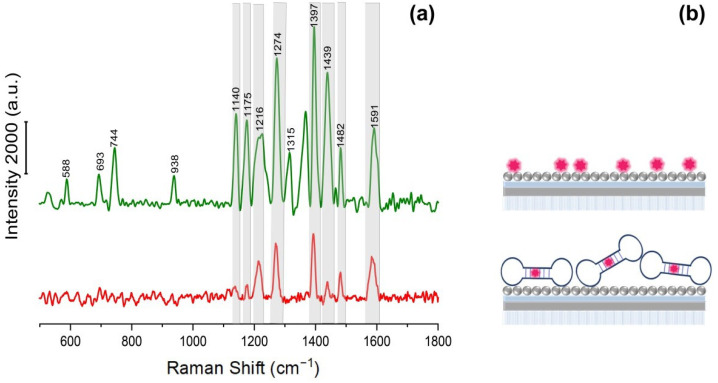
(**a**) SERS spectrum of Cy3 (green) and Cy3-aptamer mixture (red) on the obtained hybrid plasmonic substrate. (**b**) Scheme of the distribution of Cy3 and Cy3-labeled aptamer on a SERS-active hybrid structure.

Figure 4 demonstrates SERS spectra of Cy3 and the Cy3-labeled secondary aptamer. The SERS spectrum of the Cy3-labeled secondary aptamer, as well as the spectrum of pure Cy3, contains well-resolved characteristic signals on 1140, 1175, 1216, 1274, 1397, 1439, 1482, and 1591 cm^−1^, though there is a slight shift within 1–2 cm^−1^ for some peaks (Figure 4a). However, low-intensity bands in the region of small Raman shifts (588, 693, 744, 938, 1140, 1175 cm^−1^) disappear after binding an aptamer to the Cy3. Of note, the intensity of characteristic peaks (1216, 1274, 1397, 1439, 1482 cm^−1^) for the Cy3-aptamer decreased three times in comparison with Cy3. The lateral size of the aptamer is about 2–3 nm [41]. The weakening of the signal can be associated with the increased distance between the dye and the silver nanostructured surface through the primary aptamer, as schematically presented in Figure 4b. The signals at 1272 and 1394 cm^–1^ have been selected as intense characteristic bands for SERS spectra of the platform for aptasensor assays. The peak 1272 cm^−1^ corresponds to the deformation oscillation of the hydroxyl group (COH); 1394 cm^−1^ corresponds to the CN-bond symmetric stretching [42].

Further, the SERS spectra of the proposed model for aptasensor assays will be compared with the spectrum of the Cy3-aptamer. The spectrum of Sample 1 has the least similarity with the model mixture Cy3-aptamer spectrum. The spectrum retains signals at 1117, 1137, 1176, 1214, 1272, 1372, 1394, 1439, and 1584 cm^−1^. At the same time, the signal intensities (1272 and 1394 cm^−1^) of the dye in Sample 1 fall relative to the other observed peaks. There are also new modes at 1072 and 1315 cm^−1^, which may be due to a signal from an aptamer or RBD protein fragment. A significant three-fold drop in the signal for Sample 1 is associated with the enhanced distance of the dye from the SERS surface due to the RBD nanoparticle shielding. Consequently, a well-organized, specific interaction of aptasensor components with model RBD protein-covered nanoparticles aids in the reproducible SERS signal quenching from the Cy3-labeled artificial model of SARS-CoV-2 virions.

**Figure 5:**
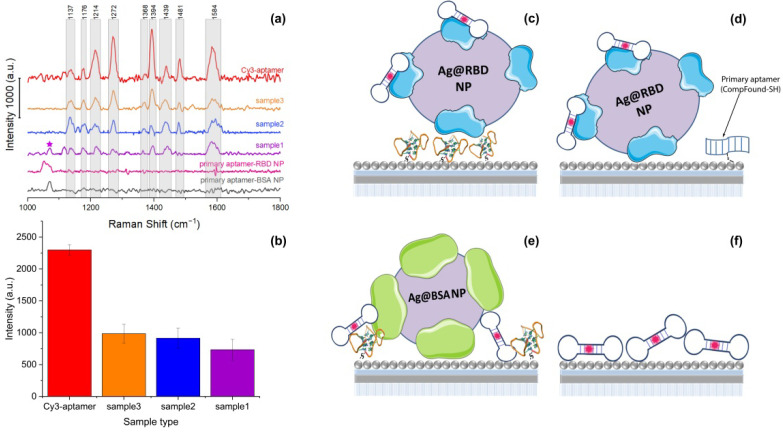
(**a**) SERS spectra of the Cy3-labeled secondary aptamer (red), Sample 3 (yellow), Sample 2 (blue), Sample 1 (violet), primary aptamer-RBD NPs (pink), primary aptamer, and BSA NPs mixture (black) on the obtained hybrid plasmonic substrate. (**b**) SERS signal at 1272 and 1394 cm^−1^ for Cy3-labeled aptamer, Sample 1, Sample 2, and Sample 3. Scheme of the distribution of (**c**) Sample 1, (**d**) Sample 2, (**e**) Sample 3, and (**f**) Cy3-labeled secondary aptamer on an SERS-active hybrid structure.

To test the selectivity of the developed model of aptasensor assay, we performed additional experiments with analytes based on the nonspecific interaction of RDB nanoparticles with the primary aptamer (further referred to as Sample 2) and the nonspecific binding of BSA nanoparticles to the secondary Cy3-labeled aptamer (further referred to as Sample 3). We investigated the dependence of signal intensity on the distance of the SERS-active label from the plasmonic surface. The similarity in the set of signals with a Cy3-aptamer probe has been observed (Figure 5a); all of the main peaks of Cy3 are present in both samples’ spectra. The last mode, associated with CH bond vibrations, can be highly intense in the spectra for Samples 2 and 3 due to the contribution of vibrations of both the dye and protein fragments and aptamers. On average, the intensity of the characteristic modes for Samples 2 and 3 is about 2.5 and 2.3 times less than the intensities for the Cy3-aptamer sample, respectively.

The signal comparison for all the samples mentioned is shown in Figure 5a,b. All the considered probes are presented in order of decreasing value of EF: from the Cy3-labeled primary aptamer to the full-size probe with specific binding RBD NPs with primary and secondary aptamers. The EF ratio for samples 1, 2, 3 is 1:1.25:1.34, respectively. The self-assembled RBD nanoparticle assay blocked the direct adsorption of the Cy3-labeled secondary aptamer on the SERS-active nanostructured surface due to the specific interaction between all components in the assay leading to strong quenching of the signal of Cy3, which agrees with previously demonstrated SERS data for a real influenza virus [24]. It is important to mention that a developed artificial indicator system gives us the opportunity to investigate simulating viruses as well as their fragments. This confirms the applicability of the proposed artificial indicator system, which does not require special permissions to work with viruses and will speed up the process of developing effective methods for their detection. The increase in signal enhancement from Sample 1 to Sample 3 may be due to the full specific binding of the developed model of SARS-CoV-2 virions with the thiol- aptamer and the Cy3-labeled aptamer (Figure 5c–f). In the case of Sample 2, the interaction of Cy3-labeled RBD NPs is not specific with the thiol-modified aptamer. It results in the direct closer contact of a labeled probe with the SERS-active surface and an increase of EF (Figure 5d). The absence of specific binding in the case of BSA NPs for Sample 3 leads to the uneven distribution of probes including BSA NPs, the labeled secondary aptamer along with the primary one as schematically presented in Figure 5e, and in summary, a further increase of the signal for Cy3 dye in comparison with Samples 1 and 2. Moreover, a comparison of SERS data of RBD protein with the Cy3-labeled secondary aptamer and Sample 1 was performed. As shown in Figure S5, no characteristic peaks of Cy3 for RBD protein with Cy3-labeled aptamer were observed. The absence of the signal can be associated with the possible dense coverage of the SERS substrate by protein fragments. As a result, the dye molecules are away from the SERS-active surface, leading to the absence of the Raman signal of Cy3.

The developed indicator system allowed us to control the distance between the SERS platform and the labeled dye moiety. Moreover, it allows us to distinguish sandwich bioassays from their fragments, which is not possible to achieve with traditional enzyme-linked immunosorbent assay (ELISA) test. The application of a fabricated efficient hybrid plasmonic structure provides detection with a sensitivity to the target virus down to 10 nM (10^10^ particles/mL). This value is slightly above the upper necessary bound of a virus in real samples. The typical SARS-CoV-2 load in the clinical samples from the patient is in the range from 10^6^ to 10^9^ virus particles/mL [43]. Thus, the analytical sensitivity of the obtained platform in our work should be further enhanced to be competitive with the limit of detection (LOD) of 10^2^–10^3^ viral particles/mL for SARS-CoV-2 for polymerase chain reaction [44,45], the LOD of 2 × 10^4^ viral particles/mL for loop-mediated isothermal amplification [46], and the LOD of 1 × 10^6^–4 × 10^8^ viral particles/mL for rapid antibody-based assays [47,48]. The developed novel plasmonic structure through varying the deposition parameters of the films and post-coating treatment paves a promising avenue to improve SERS performance of the sensor element, including the LOD, and fosters the practical application of the ultra-fast, amplification-free determination of coronaviruses.

## 4. Conclusions

Thus, we have proposed a novel SERS sensing platform based on protein-coated silver nanoparticles with a size close to the viral size as a highly stable structure for studying the detection of SARS-CoV-2 virions. Such a platform does not require special permissions to work with viruses and will speed up the process of developing effective methods for their detection. The optimal conditions for obtaining an artificial SERS-active system due to both specific and nonspecific binding of the primary aptamer and the Cy3-labeled secondary aptamer to synthetic virions were determined. Utilizing the receptor-binding domain of the S-protein of the envelope of the SARS-CoV-2 virus as a shell for model nanoparticles simulating the virus results in specific interactions by sandwich formation of it and both aptamers while bovine serum albumin results in no specific way, respectively. The developed indicator system allows us to control the distance between the fabricated efficient SERS hybrid platform and the labeled dye moiety. The ability to detect the target virus due to specific interactions with such aptameric DNA is quantitatively controlled by the degree of the quenching SERS signal from the labeled compound linked to an aptamer and paves a promising avenue to foster the practical application of the ultra-fast, amplification-free determination of coronaviruses.

## Figures and Tables

**Table 1:** Combinations of aptamers and protein-coated nanoparticles studied.

	Primary Aptamer	Nanoparticles	Secondary Aptamer	Comment
Sample 1	Found-SH	RBD NP	RBD-1C-Cy3	Experiment
Sample 2	CompFound-SH	RBD NP	RBD-1C-Cy3	Control with nonspecific primary oligonucleotide
Sample 3	Found-SH	BSA NP	RBD-1C-Cy3	Control with nonspecific protein instead of RBD

## Data Availability

Not applicable.

## Supplementary Materials

The following supporting information can be downloaded at: https://www.mdpi.com/article/10.3390/bios12090768/s1, Figure S1: Sensorgrams of 100 nM found aptamer sequence (Bt-Found), aptamer from the literature and cmpFound-Bt (nonspecific control), all carrying Bt-T10—linker at 5′(or 3′)-end; Figure S2: Reflection spectrum of SERS-active substrate; Figure S3: SERS spectrum of Cy3 (1 × 10^–6^ M). (532 nm, 0.3 mW, 5 s, 10 acc., ×40) on the obtained hybrid plasmonic substrate; Figure S4: Raman spectrum of Cy3 (1 × 10^–2^ M). (532 nm, 0.3 mW, 5 s, 10 acc., ×40) on the obtained hybrid plasmonic substrate. Figure S5: SERS spectra of Sample 1 (violet), primary aptamer-RBD protein with Cy3-labeled aptamer (magenta) (532 nm, 0.3 mW, 5 s, 10 acc., ×40).

## References

[1] Langer J., de Aberasturi D.J., Aizpurua J., Alvarez-Puebla R.A., Auguié B., Baumberg J.J., Bazan G.C., Bell S.E.J., Boisen A., Brolo A.G. (2020). Present and Future of Surface-Enhanced Raman Scattering. ACS Nano.

[2] Cao H., Cao H., Li Y., Sun Z., Yang Y., Jiao T. (2022). A Novel Natural Surface-Enhanced Fluorescence System Based on Reed Leaf as Substrate for Crystal Violet Trace Detection. Chinese Phys. B.

[3] Samodelova M.V., Kapitanova O.O., Evdokimov P.V., Eremina O.E., Goodilin E.A., Veselova I.A. (2022). Plasmonic Features of Free-Standing Chitosan Nanocomposite Film with Silver and Graphene Oxide for SERS Applications. Nanotechnology.

[4] Lenzi E., De Jimenez Aberasturi D., Henriksen-Lacey M., Piñeiro P., Muniz A.J., Lahann J., Liz-Marzán L.M. (2022). SERS and Fluorescence-Active Multimodal Tessellated Scaffolds for Three-Dimensional Bioimaging. ACS Appl. Mater. Interfaces.

[5] Lu Y., Dong W., Chen Z., Pors A., Wang Z., Bozhevolnyi S.I. (2016). Gap-Plasmon Based Broadband Absorbers for Enhanced Hot-Electron and Photocurrent Generation. Sci. Rep..

[6] Nielsen M.G., Gramotnev D.K., Pors A., Albrektsen O., Bozhevolnyi S.I. (2011). Continuous Layer Gap Plasmon Resonators. Opt. Express.

[7] Tatmyshevskiy M.K., Yakubovsky D.I., Kapitanova O.O., Solovey V.R., Vyshnevyy A.A., Ermolaev G.A., Klishin Y.A., Mironov M.S., Voronov A.A., Arsenin A.V. (2021). Hybrid Metal-Dielectric-Metal Sandwiches for SERS Applications. Nanomaterials.

[8] Novikov S.M., Boroviks S., Evlyukhin A.B., Tatarkin D.E., Arsenin A.V., Volkov V.S., Bozhevolnyi S.I. (2020). Fractal Shaped Periodic Metal Nanostructures Atop Dielectric-Metal Substrates for SERS Applications. ACS Photonics.

[9] Kasera S., Herrmann L.O., Del Barrio J., Baumberg J.J., Scherman O.A. (2014). Quantitative Multiplexing with Nano-Self-Assemblies in SERS. Sci. Rep..

[10] Pisarev E.K., Kapitanova O.O., Vesolova I.A., Zvereva M.I. (2021). Amplification-Free Identification and Determination of Nucleic Acids by Surface Plasmon Resonance and Surface-Enhanced Raman Spectroscopy. Mosc. Univ. Chem. Bull..

[11] Tadesse L.F., Safir F., Ho C.S., Hasbach X., Khuri-Yakub B.P., Jeffrey S.S., Saleh A.A.E., Dionne J. (2020). Toward Rapid Infectious Disease Diagnosis with Advances in Surface-Enhanced Raman Spectroscopy. J. Chem. Phys..

[12] Saviñon-Flores F., Méndez E., López-Castaños M., Carabarin-Lima A., López-Castaños K.A., González-Fuentes M.A., Méndez-Albores A. (2021). A Review on Sers-Based Detection of Human Virus Infections: Influenza and Coronavirus. Biosensors.

[13] Ambartsumyan O., Gribanyov D., Kukushkin V., Kopylov A., Zavyalova E. (2020). SERS-Based Biosensors for Virus Determination with Oligonucleotides as Recognition Elements. Int. J. Mol. Sci..

[14] Paria D., Kwok K.S., Raj P., Zheng P., Gracias D.H., Barman I. (2022). Label-Free Spectroscopic SARS-CoV-2 Detection on Versatile Nanoimprinted Substrates. Nano Lett..

[15] Payne T.D., Klawa S.J., Jian T., Kim S.H., Papanikolas M.J., Freeman R., Schultz Z.D. (2021). Catching COVID: Engineering Peptide-Modified Surface-Enhanced Raman Spectroscopy Sensors for SARS-CoV-2. ACS Sensors.

[16] Abdullah M.B., Dab C., Almalki M., Alnaim A., Abuzir A., Awada C. (2022). Ultrafast Detection of SARS-CoV-2 Spike Protein (S) and Receptor-Binding Domain (RBD) in Saliva Using Surface-Enhanced Raman Spectroscopy. Appl. Sci..

[17] Sarychev A.K., Sukhanova A., Ivanov A.V., Bykov I.V., Bakholdin N.V., Vasina D.V., Gushchin V.A., Tkachuk A.P., Nifontova G., Samokhvalov P.S. (2022). Label-Free Detection of the Receptor-Binding Domain of the SARS-CoV-2 Spike Glycoprotein at Physiologically Relevant Concentrations Using Surface-Enhanced Raman Spectroscopy. Biosensors.

[18] Ye J., Yeh Y.-T., Xue Y., Wang Z., Zhang N., Liu H., Zhang K., Ricker R., Yu Z., Roder A. (2022). Accurate Virus Identification with Interpretable Raman Signatures by Machine Learning. Proc. Natl. Acad. Sci. USA.

[19] Shanmukh S., Jones L., Driskell J., Zhao Y., Dluhy R., Tripp R.A. (2006). Rapid and Sensitive Detection of Respiratory Virus Molecular Signatures Using a Silver Nanorod Array SERS Substrate. Nano Lett..

[20] Lee J.H., Kim B.C., Oh B.K., Choi J.W. (2015). Rapid and Sensitive Determination of HIV-1 Virus Based on Surface Enhanced Raman Spectroscopy. J. Biomed. Nanotechnol..

[21] Jen Lin Y. (2014). A Rapid and Sensitive Early Diagnosis of Influenza Virus Subtype via Surface Enhanced Raman Scattering. J. Biosens. Bioelectron..

[22] Kukushkin V.I., Ivanov N.M., Novoseltseva A.A., Gambaryan A.S., Yaminsky I.V., Kopylov A.M., Zavyalova E.G. (2019). Highly Sensitive Detection of Influenza Virus with SERS Aptasensor. PLoS ONE.

[23] Ebrem Bilgin B., Torun H., Ilgü M., Yanik C., Batur S.N., Çelik S., Öztürk M., Dogan Ö., Ergönül Ö., Solaroglu I. (2022). Clinical Validation of SERS Metasurface SARS-CoV-2 Biosensor.

[24] Chen H., Park S.G., Choi N., Moon J.I., Dang H., Das A., Lee S., Kim D.G., Chen L., Choo J. (2020). SERS Imaging-Based Aptasensor for Ultrasensitive and Reproducible Detection of Influenza Virus A. Biosens. Bioelectron..

[25] Chen H., Park S.K., Joung Y., Kang T., Lee M.K., Choo J. (2022). SERS-Based Dual-Mode DNA Aptasensors for Rapid Classification of SARS-CoV-2 and Influenza A/H1N1 Infection. Sens. Actuators B Chem..

[26] Lim W.Y., Lan B.L., Ramakrishnan N. (2021). Emerging Biosensors to Detect Severe Acute Respiratory Syndrome Coronavirus 2 (SARS-CoV-2): A Review. Biosensors.

[27] Tabrizi M.A., Acedo P. (2022). An Electrochemical Impedance Spectroscopy-Based Aptasensor for the Determination of SARS-CoV-2-RBD Using a Carbon Nanofiber–Gold Nanocomposite Modified Screen-Printed Electrode. Biosensors.

[28] Qu J.H., Leirs K., Maes W., Imbrechts M., Callewaert N., Lagrou K., Geukens N., Lammertyn J., Spasic D. (2022). Innovative FO-SPR Label-Free Strategy for Detecting Anti-RBD Antibodies in COVID-19 Patient Serum and Whole Blood. ACS Sens..

[29] Calvo-Lozano O., Sierra M., Soler M., Estévez M.C., Chiscano-Camón L., Ruiz-Sanmartin A., Ruiz-Rodriguez J.C., Ferrer R., González-López J.J., Esperalba J. (2022). Label-Free Plasmonic Biosensor for Rapid, Quantitative, and Highly Sensitive COVID-19 Serology: Implementation and Clinical Validation. Anal. Chem..

[30] Besselink G., Schütz-trilling A., Veerbeek J., Verbruggen M., Van Der Meer A. (2022). Asymmetric Mach—Zehnder Interferometric Biosensing for Quantitative and Sensitive Multiplex Detection of Anti-SARS-CoV-2 Antibodies in Human Plasma. Biosensors.

[31] Svobodova M., Skouridou V., Jauset-Rubio M., Viéitez I., Fernández-Villar A., Cabrera Alvargonzalez J.J., Poveda E., Bofill C.B., Sans T., Bashammakh A. (2021). Aptamer Sandwich Assay for the Detection of SARS-CoV-2 Spike Protein Antigen. ACS Omega.

[32] Chen H., Park S.G., Choi N., Kwon H.J., Kang T., Lee M.K., Choo J. (2021). Sensitive Detection of SARS-CoV-2 Using a SERS-Based Aptasensor. ACS Sens..

[33] Zhang D., Zhang X., Ma R., Deng S., Wang X., Wang X., Zhang X., Huang X., Liu Y., Li G. (2021). Ultra-Fast and Onsite Interrogation of Severe Acute Respiratory Syndrome Coronavirus 2 (SARS-CoV-2) in Waters via Surface Enhanced Raman Scattering (SERS). Water Res..

[34] Khrenova M.G., Nikiforova L., Grabovenko F., Orlova N., Sinegubova M. (2022). In Vitro Selection of an Aptamer Targeting SARS-CoV-2 Spike Protein with Nanopore Sequence Identification Reveals Discrimination Between the Authentic Strain and Omicron. ChemRxiv.

[35] Song Y., Song J., Wei X., Huang M., Sun M., Zhu L., Lin B., Shen H., Zhu Z., Yang C. (2020). Discovery of Aptamers Targeting the Receptor-Binding Domain of the SARS-CoV-2 Spike Glycoprotein. Anal. Chem..

[36] Sinegubova M.V., Orlova N.A., Kovnir S.V., Dayanova L.K., Vorobiev I.I. (2021). High-Level Expression of the Monomeric SARS-CoV-2 S Protein RBD 320-537 in Stably Transfected CHO Cells by the EEF1A1-Based Plasmid Vector. PLoS ONE.

[37] Novikov S.M., Streletskiy O.A., Doroshina N.V., Yakubovsky D.I., Mironov M.S., Sychev V.V., Voronov A.A., Arsenin A.V., Volkov V.S. (2021). Long-Term Stable Structures Formed by Ion-Beam Modification of Silver Film for SERS Applications. J. Phys. Conf. Ser..

[38] Waghmare M., Khade B., Chaudhari P., Dongre P. (2018). Multiple Layer Formation of Bovine Serum Albumin on Silver Nanoparticles Revealed by Dynamic Light Scattering and Spectroscopic Techniques. J. Nanoparticle Res..

[39] Grabovenko F., Nikiforova L., Yanenko B., Ulitin A., Loktyushov E., Zatsepin T., Zavyalova E., Zvereva M. (2022). Glycosylation of Receptor Binding Domain of SARS-CoV-2 S-Protein Influences on Binding to Immobilized DNA Aptamers. Int. J. Mol. Sci..

[40] Le Ru E.C., Blackie E., Meyer M., Etchegoint P.G. (2007). Surface Enhanced Raman Scattering Enhancement Factors: A Comprehensive Study. J. Phys. Chem. C.

[41] Eremina O.E., Zatsepin T.S., Farzan V.M., Veselova I.A., Zvereva M.I. (2020). DNA Detection by Dye Labeled Oligonucleotides Using Surface Enhanced Raman Spectroscopy. Mendeleev Commun..

[42] Jaworska A., Pyrak E., Kudelski A. (2019). Comparison of the Efficiency of Generation of Raman Radiation by Various Raman Reporters Connected via DNA Linkers to Different Plasmonic Nano-Structures. Vib. Spectrosc..

[43] Despres H.W., Mills M.G., Shirley D.J., Schmidt M.M., Huang M.L., Roychoudhury P., Jerome K.R., Greninger A.L., Bruce E.A. (2022). Measuring Infectious SARS-CoV-2 in Clinical Samples Reveals a Higher Viral Titer:RNA Ratio for Delta and Epsilon vs. Alpha Variants. Proc. Natl. Acad. Sci. USA.

[44] Kim H.N., Yoon S.Y., Lim C.S., Yoon J. (2022). Comparison of Three Molecular Diagnostic Assays for SARS-CoV-2 Detection: Evaluation of Analytical Sensitivity and Clinical Performance. J. Clin. Lab. Anal..

[45] Yang J., Han Y., Zhang R., Zhang R., Li J. (2021). Comparison of Analytical Sensitivity of SARS-CoV-2 Molecular Detection Kits. Int. J. Infect. Dis..

[46] Yan C., Cui J., Huang L., Du B., Chen L., Xue G., Li S., Zhang W., Zhao L., Sun Y. (2020). Rapid and Visual Detection of 2019 Novel Coronavirus (SARS-CoV-2) by a Reverse Transcription Loop-Mediated Isothermal Amplification Assay. Clin. Microbiol. Infect..

[47] Chan K.H., To K.K.W., Chan J.F.W., Li C.P.Y., Chen H., Yuen K.Y. (2013). Analytical Sensitivity of Seven Point-of-Care Influenza Virus Detection Tests and Two Molecular Tests for Detection of Avian Origin H7N9 and Swine Origin H3N2 Variant Influenza a Viruses. J. Clin. Microbiol..

[48] Peters T.R., Blakeney E., Vannoy L., Poehling K.A. (2013). Evaluation of the Limit of Detection of the BD VeritorTM System Flu A+B Test and Two Rapid Influenza Detection Tests for Influenza Virus. Diagn. Microbiol. Infect. Dis..

