# Supplementary material for: Model of the SARS-CoV-2 Virus for Development of a DNA-Modified, Surface-Enhanced Raman Spectroscopy Sensor with a Novel Hybrid Plasmonic Platform in Sandwich Mode
Source: Biosensors (Basel). 2022 Sep 19;12(9):768. doi: 10.3390/bios12090768 (PMC9497064; doi:10.3390/bios12090768)
Supplement: Supplementary file 1 [file biosensors-12-00768-s001.zip › biosensors-1855255-supplementary.pdf]

Supplementary Information

Model of the SARS-CoV-2 Virus for Development of a DNA-Modified, Surface-Enhanced Raman Spectroscopy Sensor with a Novel Hybrid Plasmonic Platform in Sandwich Mode

Mariia V. Samodelova [1], Olesya O. Kapitanova [1*], Nadezda F. Meshcheryakova [1], Sergey. M. Novikov [2], Nikita R. Yarenkov [1], Oleg A. Streletskii [3], Dmitry I. Yakubovsky [2], Fedor I. Grabovenko [1], Gleb A. Zhdanov [1], Aleksey V. Arsenin [2], Valentyn S. Volkov [2], Elena G. Zavyalova [1], Irina A. Veselova [1], Maria I. Zvereva [1]

[1] Faculty of Chemistry, Lomonosov Moscow State University, Leninskie gory 1, Moscow, 119991, Russia
[2] Center for Photonics and 2D Materials, Moscow Institute of Physics and Technology, 141700 Dolgoprudny, Russia
[3] Faculty of Physics, Lomonosov Moscow State University, Leninskie gory 1, Moscow, 119991, Russia
* Correspondence: olesya.kapitanova@gmail.com

Aptamer preparation and binding experiments

Commercially available reagents were used to synthesize oligonucleotides via a solid-phase phosphonamidite method, followed by high performance liquid chromatography (HPLC) purification.

To provide proper folding, aptamer stock solutions (2 μM in PBS buffer with 0.55 mM $MgCl_2$) were preheated at 95 °C for 5 min, then cooled down to 0 °C on ice for 5 min and, finally, was kept at room temperature for 20 min.

The stock solution of the aptamer was diluted with 5X binding buffer and distilled water to obtain the final concentration of 200 nM aptamer in 1X binding buffer (BB_{BSA}: 1X PBS buffer, 0.55 mM $MgCl_2$, 0.1 mg/mL BSA, 0.002% Tween-20). To test the binding specificity, the experiments with biotinylated 41 bp ssDNA with aptamer-complementary sequence: 5'- GGTGCTGTTCGGATTCTATCGTGTTTCCCTA-$(T)_{10}$-3'- biotin were conducted.

All the experiments were carried out at room temperature. BLItz (ForteBio, Fremont, CA, USA) instrument in advanced kinetics mode with shaking at 2200 RPM. Streptavidin biosensors (ForteBio, Fremont, CA, USA) were hydrated in a binding buffer for 10 min prior to the experiment. Each measurement step was carried out in black tubes (Sigma-Aldrich, New York, NY, USA) with at least 300 μL of a respective solution. The optimized BLI protocol was split in two parts for handiness and comprised the following steps: 1) oligonucleotide binding step: initial baseline carried in binding buffer for 60 s; loading of aptamer for 210 s; the second baseline in binding buffer for 60 s; 2) protein binding step: baseline carried in binding buffer for 30 s; association for 130 s (50, 100 or 200 nM protein solution in binding buffer); dissociation in binding buffer for 130 s; washing step-1 carried in 1 M ethanolamine (pH 8.3) for 120 s; washing step-2 carried in binding buffer for 120 s.

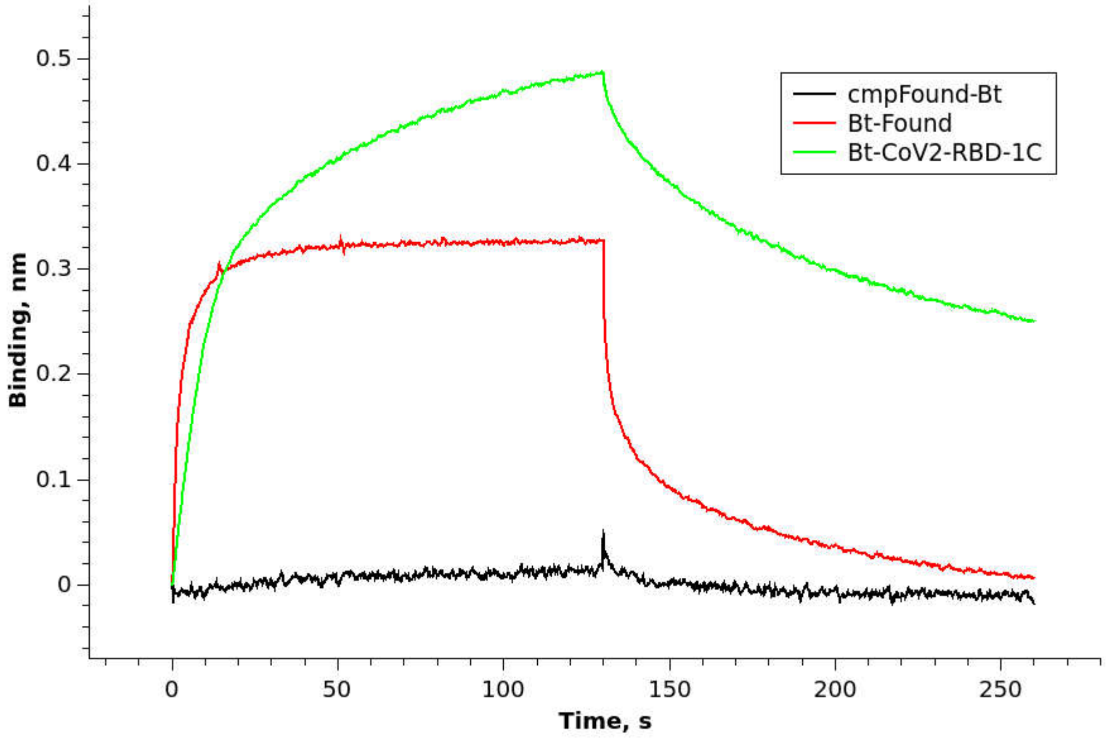

Figure S1. Sensorgrams of 100 nM found aptamer sequence (Bt-Found), aptamer from the literature [35] and cmpFound-Bt (nonspecific control), all carrying Bt-T10 - linker at 5′(or 3′)-end.

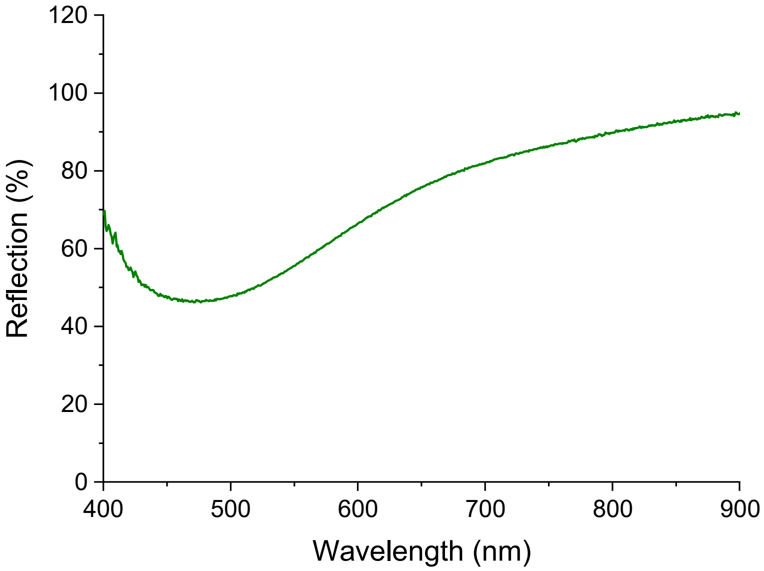

Figure S2. Reflection spectrum of SERS-active substrate.

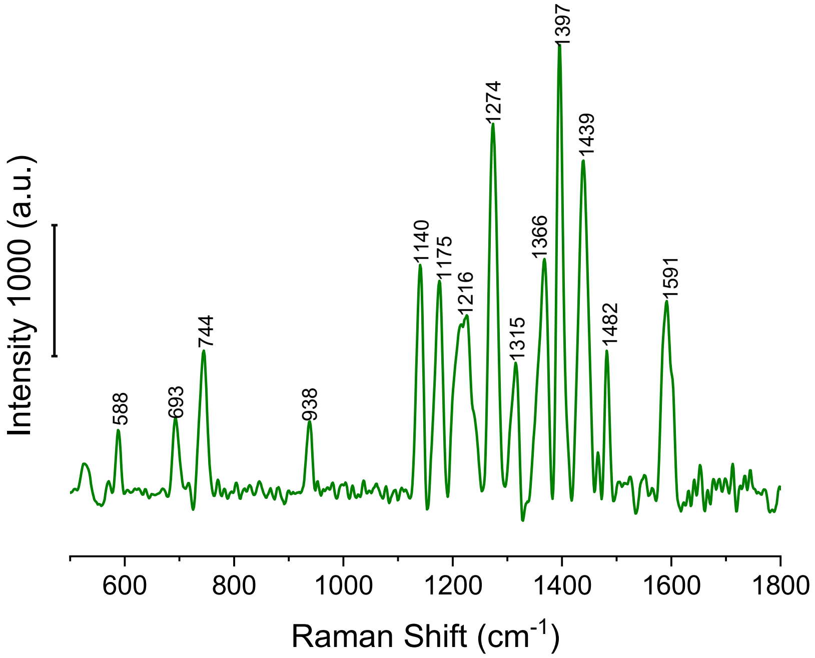

Figure S3. SERS spectrum of Cy3 ($1\cdot10^{-6}$ M). (532 nm, 0.3 mW, 5 s, 10 acc., x 40) on the obtained hybrid plasmonic substrate.

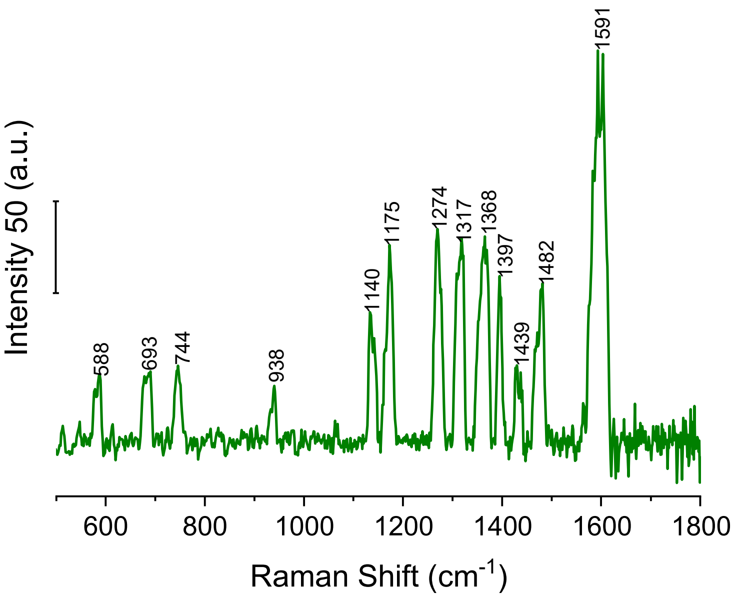

Figure S4. Raman spectrum of Cy3 ($1\cdot10^{-2}$ M). (532 nm, 0.3 mW, 5 s, 10 acc., x 40) on the obtained hybrid plasmonic substrate.

The relative standard deviation (RSD) was calculated according to the following equation:

$$RSD = \frac{S_x}{\bar{x}} \cdot 100\%$$

where S_x is the standard deviation and $\bar{x}$ is the average value of the enhancement factor (EF).

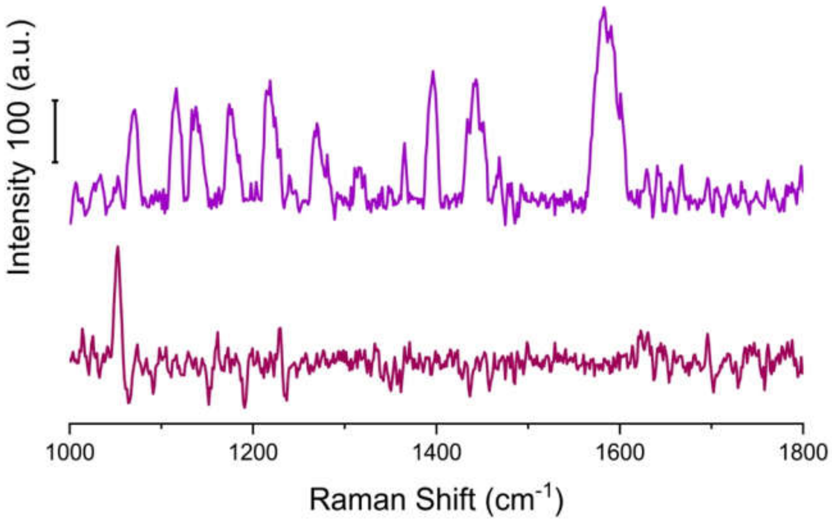

Figure S5. SERS spectra of Sample 1 (violet), primary aptamer-RBD protein with Cy3-labeled aptamer (magenta) (532 nm, 0.3 mW, 5 s, 10 acc., x 40).

Reference

35. Song, Y.; Song, J.; Wei, X.; Huang, M.; Sun, M.; Zhu, L.; Lin, B.; Shen, H.; Zhu, Z.; Yang, C. Discovery of Aptamers Targeting the Receptor-Binding Domain of the SARS-CoV-2 Spike Glycoprotein. Anal. Chem. 2020, 92, 9895–9900, doi:10.1021/acs.analchem.0c01394.
